# A Global Reassessment of Solitary-Sociable Dolphins

**DOI:** 10.3389/fvets.2018.00331

**Published:** 2019-01-22

**Authors:** Laetitia Nunny, Mark P. Simmonds

**Affiliations:** ^1^Wild Animal Welfare, Barcelona, Spain; ^2^School of Veterinary Science, University of Bristol, Bristol, United Kingdom; ^3^Humane Society International, London, United Kingdom

**Keywords:** bottlenose dolphin, solitary dolphin, sociable dolphin, lone dolphin, solitary-sociable dolphin, beluga, animal welfare

## Abstract

Dolphins are typically regarded as highly social animals. However, some individuals live apart from their own species and may come to socialize with people through a recognized series of stages which are presented and expanded on in this paper. The term “solitary-sociable dolphins” has been used to describe these animals and such individuals have been identified from several different species and reported in many parts of the world. In many instances, the interactions with people that may follow their original isolation, and which typically become more intense over time, have created situations where the welfare of the animal has been compromised by disturbance, injury, the feeding of inappropriate items and aggressive human behavior. Several solitary-sociable dolphins have also been deliberately injured and killed by humans. People who interact with these dolphins may also put themselves at risk of injury. This paper reports on recent cases drawing on published and unpublished sources. Since 2008, 32 solitary dolphins have been recorded including 27 bottlenose dolphins (25 *Tursiops truncatus* and two *Tursiops aduncus*), two striped dolphins and three common dolphins. Four solitary belugas have also been recorded. There are some ten solitary dolphins and one beluga known at the present time. Laws and guidelines currently in place to protect solitary-sociable dolphins need to be strengthened and interactions with people should be avoided or, at the least, carefully managed to protect both the dolphin and the humans involved in the interaction. Terms, such as disturbance and harassment which are included in laws need to be clearly defined. Additionally, management plans for solitary-sociable dolphins need to be developed and adapted on a case by case basis taking into account the individual dolphin's sex, age, personality, stage of sociability and home range. It is also important that government officials and local stakeholders work together to implement guidelines which set out how the public can observe or interact with the dolphin safely.

## Introduction

The phenomenon of “solitary-sociable dolphins” has been described by various authors and cases have been reported from all over the world and across many decades [e.g., (1–3)]. The vast majority of such animals are bottlenose dolphins (*Tursiops truncatus* and, to a lesser extent, *Tursiops aduncus*) possibly because, as predominantly inshore coastal species, they are more likely to come into contact with humans than other dolphins (1, 4). This behavior has also been reported in belugas (*Delphinapterus leucas*), narwhals (*Monodon monoceros*), orcas (*Orcinus orca*), tucuxis (*Sotalia fluviatilis*) and other dolphin species, including common (*Delphinus delphis*), striped (*Stenella coeruleoalba*), dusky (*Lagenorhynchus obscurus*), Risso's (*Grampus griseus*) and pantropical spotted (*Stenella attenuata*) dolphins (5).

Wilke et al. (2) described these animals as dolphins “who have little or no contact with conspecifics and who regularly closely approach humans, often including touch, social, sexual and play behaviors.” Müller and Bossley (6) suggested that it is likely that there are different “types” of solitary dolphins exhibiting different behaviors and that a single definition for these animals would be simplistic and confusing. Wilke et al. (2) proposed a number of stages that an individual animal passes through as he or she changes behavior from being simply “solitary” to being a “friendly solitary.” Using these stages, we categorize recent cases and also propose two new additional stages.

Interactions between wild dolphins and humans have been increasing around the world often encouraged by local tourist agencies (7) and, as solitary-sociable dolphins often restrict their movements to a small area, they may be relatively easy for the public to access (6). Human-dolphin interactions may lead to various management and welfare problems and even the death of the animal and it is, therefore, appropriate that consideration is given to the management and protection of such dolphins (3) and also the safety of humans entering the water with them.

In this paper we review current knowledge regarding solitary-sociable delphinids and monodontids and detail cases recorded since 2008. We consider the stages that they can pass through as they become increasingly sociable and how to improve the protection afforded to them.

## Methods

We sought the most recent information about solitary-sociable dolphins by (i) sending a request for information via the MARMAM online mailing list, (ii) conducting a variety of internet searches, including using academic databases and (iii) contacting those involved in monitoring their local dolphin populations. We also advertised this work as ongoing at the 2018 meeting of the Scientific Committee of the International Whaling Commission.

The information gathered is displayed in two tables; one for animals recorded in Europe and one for animals from other parts of the world. Criteria for inclusion of an animal included that it had to belong to the family of delphinids or monodontids and that it had to have been seen since 2008. Any dolphins that died or disappeared before 2008 are not included in our tables. The animal also had to have been recorded on its own for a prolonged period (at least a few weeks). Then we considered the behaviors exhibited and assigned the dolphins to one of the stages (and, if possible, levels) of sociability described below in section Stages of Sociability. Wherever possible we contacted scientists and other observers who had reported the animals to verify information.

The solitary belugas are not assigned to any of the stages of sociability here as it is not clear whether their behavior develops in the same way as it does for dolphins (C. Kinsman 2018, pers. comm., 9 November). This is an area that merits future research as in Canada there have been an increasing number of solitary belugas reported in recent years (see **Table 3**), and this year there has also been a case of a solitary beluga in the waters off the United Kingdom (see section Cases of Solitary-Sociable Dolphins Since 2008).

## Why And How Dolphins Become Solitary And Sociable

Bottlenose dolphin society is described by Müller and Bossley (6) as “a complex mixture of associations” and solitary individuals may be considered at one end of the range of observed sociability. Group sizes may range from one to over 100 and may be influenced by habitat structure and activity patterns (12). Bottlenose dolphins typically live in a “fission-fusion” society and group composition changes continuously and frequently (12–14). Food availability and loss of habitat may sometimes determine dolphin social structures and could lead to solitary behavior (4, 6). Different factors, or a combination of factors, may prompt a dolphin to become solitary for a prolonged period and it is important to note that such an animal will not necessarily start interacting or socializing with humans.

Differences between individuals in terms of their behavioral choices could mean that some animals are more likely to become solitary than others (6). Connor et al. (13) reported that in Sarasota Bay, Florida and Shark Bay, Western Australia some female bottlenose dolphins are more solitary whilst others are more social. Some dolphins may become solitary because of their individual life experience. Thus, the death of a male dolphin's coalition partner, the loss of a whole group or a mother (due to illness, bycatch or hunting) or the poor health of an individual because of illness or injury could all potentially lead to a dolphin becoming solitary (6). In Brazil, “Viola” the solitary tucuxi allowed humans to touch it after its mother was killed by a fisherman (15). In Mexico, “Pechocho” the bottlenose dolphin is also believed to have become solitary after his mother died.^1^ Solitariness could, therefore, in some cases, be a response to trauma (6). It is also possible that young dolphins that have not learned the necessary social skills from their mother have problems integrating into dolphin society.

The majority of recorded cases of solitary dolphins come from areas close to the coast, perhaps because the open ocean is a more dangerous place for a lone animal or because they are more likely to be observed and recorded when they are in coastal areas (6). The available evidence indicates that, fairly obviously, for a solitary dolphin to become a solitary-sociable dolphin, it has to be in an area where it can come into frequent contact with people.

Many of the reported cases of solitary dolphins come from Europe (see Tables 1, 2) and Müller and Bossley (6) cite the past decimation of various dolphin populations in Europe as a factor which could contribute to the increased likelihood of a dolphin becoming solitary. They suggest that, in the past, dolphin social groups overlapped and so an individual that was leaving one group, could easily find another group to join. In areas where numbers have been reduced, social groups may be more separated and so an individual may find itself alone for a longer period before it encounters another group and it could, potentially, become solitary during this period. Simmonds and Stansfield (21) proposed that, in the United Kingdom, an increasing number of solitary bottlenose dolphins could be due to the distance between the relatively few remaining groups, and that individuals which disperse or are displaced from their natal group may end up on their own because they simply do not find another group to join.

**Table 1:** Solitary-sociable dolphins 109AD−2008 by region and species [adapted from Goodwin and Dodds (5)].

**Region**	**Bottlenose dolphin (*Tursiops truncatus* and *Tursiops aduncus*)**	**Other species**
Europe, Middle East, and North Africa	39	3 (species unknown)
South Africa	2	0
Caribbean and Americas	10	20 (13 beluga, 3 orca, 1 narwhal, 1 pantropical spotted dolphin, 1 tucuxi, 1 species unknown)
Australia and New Zealand	9	6 (4 Common dolphin, 1 Dusky dolphin, 1 Risso's dolphin)
Asia	2	0
Total	62	29

**Table 2:** Solitary and solitary-sociable dolphins in Europe since 2008.

**Stage (Level) reached**	**Name of dolphin**	**Location**	Species^*^	**Gender**	**First seen**	**Last seen / date of death**	**References**
1	Unnamed	Monfalcone, Italy	Dd	Presumed F	Jun. 2010	Aug. 2011	(16)
1	Stormy	Wales, UK	Dd	?	Dec. 2014	Apr. 2015	^4^
3 or 4 (2 or 3)	Kylie / Colin / Donna	Firth of Clyde, Scotland, UK	Dd	F	Approximately 2001	Present	(17); M. Cosentino, 2018, pers. comm., 2 June.; ^5^
–	Benny	Thames Estuary, UK	Dl	?	July, 2018	Present	^6^^,^ ^7^
1 or 2	SC1	Vinodol Channel, Croatia	Sc	F	Aug. 2004	Last seen Jul. 2009	(18)
1	?	Mali Lošinj harbor, Croatia	Sc	?	Aug. 2008	Last seen 11 Sept. 2008	(19)
1 or 2	Rudolf	Ostend and Nieuwpoort, Belgium	Tt	?	2007	Last seen 2008 (or possibly 2010)	(20); ^8^
2	? (possibly Rudolf)	Knokke-Heist, Belgium	Tt	?	Jul. 2010	Last seen Oct. 2010	(20)
4 (4)	Bobi	Karin Sea, Croatia	Tt	M	Apr. 2014	Last seen 2016 (or possibly 2017)	^9^^,^ ^10^^,^ ^11^^,^ ^12^
2	? (possibly Bobi)	Slano, Croatia	Tt	?	Jun. 2017	Seen a few times. Possibly still present	D. Crljen, 2018, pers. comm. 18 June;^12^
4 (5)	Dusty / Sandy / Mara	North Clare / Inis Oírr, Ireland	Tt	F	2000	Present	R. Meade, 2018, pers. comm. 7 May; ^13^^,^ ^14^^,^ ^15^^,^ ^16^^,^ ^34^^,^ ^64^
1	Nimmo / Salty	Galway, Ireland	Tt	?	Since at least 2008	Present	^17^
2	Doogie / Dougie	Tory Island, Ireland	Tt	F	2006	Last seen? 2008?	(5); ^18^^,^ ^19^^,^ ^64^
4 (3)	Fungie	Dingle, Ireland	Tt	M	1983	Present	^20^
2 (5)	Clet / Nick / Nobby / George II / Hobnob	France, UK, Ireland	Tt	M	2008	Last seen summer 2015	^2^^,^ ^21^^,^ ^22^^,^ ^54^
1	Fiete / Freddy	Brittany, France and Kiel, Germany	Tt	M	2016	Aug. 2017	M. Perri, 2018, pers. comm., 18 May; ^3^^,^ ^23^
2	Gaspar / Jean Copo'h / Jean Floc'h	Brittany, France and Galicia, Spain	Tt	M	2003	Last seen 2010	^24^^,^ ^25^^,^ ^26^
1	Lilou / Wifi	Brittany, France	Tt	M	2007	?	^27^^,^ ^28^
4	Randy / Dony / Georges	UK, Ireland, France, Holland, Belgium.	Tt	M	April 2001	Present (Currently in Brittany, France)	^29^^,^ ^30^
3 (3 or 4)	Elcano	Northern Spain, Western France	Tt	M	Feb. 2013	Last seen Sept. 2013	^31^^,^ ^32^
3 or 4 (5)	Zafar / Toto	Brittany, France	Tt	?	Jun. 2017	Present	^61^^,^ ^33^
0	?	Portsmouth and Isle of Wight, UK	Tt	?	Jun. 2017	?	^29^
3 or 4 (2)	Splashy	Cornwall, UK	Tt	M	Jul. 2017	Last seen Mar. 2018	D. Jarvis 2018, pers. comm., 29 May; A. Lowe 2018, pers. comm., 16 March

* *Dd, short-beaked common dolphin (Delphinus delphis); Dl, Beluga (Delphinapterus leucas); Sc, striped dolphin (Stenella coeruleoalba); Tt, bottlenose dolphin (Tursiops truncatus); Ta, Indo-Pacific bottlenose dolphin (Tursiops aduncus)*.

Lockyer and Müller (4) stated that the time it takes for a dolphin to become sociable (meaning here that it readily interacts with people in the water) depends, in part, on the frequency of interactions and the patience and determination of the humans who interact with the animal as well as the age of the dolphin, whether or not it has experienced aggression from humans in the past, and its personality.

### Stages of Sociability

Whatever the reason for the dolphin's initial solitariness, its subsequent development into a “sociable” dolphin that interacts with humans happens through a process which has been described by Wilke et al. (2), who identified four stages:

Stage 1: a solitary dolphin establishes itself in a limited home range. The area has sufficient prey and an area where the dolphin likes to rest, such as next to a buoy or moored boat. The dolphin may follow boats or approach fishing gear but it does not approach humans.
Stage 2: The dolphin may start to follow boats more regularly and to engage in bow-riding as well as investigating ropes, chains, buoys etc. The dolphin is interested in people who enter the water to swim with it, but it maintains a distance.
Stage 3: The dolphin becomes accustomed to one or more people who have deliberately tried to habituate it. Humans swim and dive with the dolphin, touch it and even hold its dorsal fin so that they can be pulled through the water. The dolphin may initiate some of these interactions and thereby help the habituation process.
Stage 4: Thanks to media reports, the dolphin becomes a tourist attraction. People come from further afield to see and swim with the dolphin. The dolphin may start to exhibit dominant, aggressive and sexual behaviors.

Wilke et al. (2) suggest that some solitary dolphin cases only progress to Stage 2 or Stage 3. While some dolphins allow human contact quite quickly, for others it takes more time before they will accept interactions and touching in the water and such habituation requires considerable effort from the humans initiating it (22).

It is also possible that a dolphin may turn up in a new location or a new part of its home range in a condition in which he or she is already “friendly” toward humans due to experiences in its previous location (2). Indeed, Doak (23) noted that whereas some solitary-sociable dolphins have very limited home ranges (e.g., “Fungie” in the Dingle area of Ireland), others range more widely or move their home bases on occasion. In the more recent cases of solitary dolphins, “Clet” and “Fiete” traveled great distances and did not demonstrate site fidelity for extended periods. “Clet” was first seen in Brittany (France) before being recorded in Cornwall, Wales, the Isle of Man and Scotland.^2^ “Fiete,” meanwhile, traveled over 2,000 km from Kiel (Germany) to Saint-Malo in Brittany in a period of 2 months.^3^

Wilke (2007), cited in Goodwin (14), suggested developing Stages 3 and 4 further to include the following levels:

Level 1: the dolphin only interacts with boats during its sociable period,
Level 2: the dolphin interacts with humans but does not allow direct physical contact,
Level 3: the dolphin allows direct contact but usually only with certain people,
Level 4: the dolphin allows direct contact with anyone. It does not demonstrate socio-sexual or dominance behaviors,
Level 5: the dolphin allows direct contact with anyone and regularly exhibits socio-sexual and dominance behaviors.

Goodwin and Dodds (5) proposed two further stages. They suggested that a Stage 5 dolphin would continue to interact with humans and boats but would also spend more time engaged in other types of interactions including with other cetacean species, seals and/or birds. We consider that interactions with other species could happen during a number of the stages e.g., “Kylie” the solitary-sociable common dolphin in the Firth of Clyde, Scotland is in Stage 3 or 4 but is sometimes sighted with a harbor porpoise (*Phocoena phocoena*) (17). We do not see this as a discretely separate stage. Goodwin and Dodds (5) also proposed a Stage 6 where the dolphin begins to interact with its own species again.

## Results

### Cases of Solitary-Sociable Dolphins Since 2008

There are many cases throughout history of solitary-sociable dolphins. Table 1 provides a summary of animals up until 2008 and is based on an earlier inventory (5). This table may not be complete but it gives some idea of the numbers concerned. Of the 91 animals listed, 62 were recorded as bottlenose dolphins [61 *T. truncatus* and one *T. aduncus*, noting that until 1998 all bottlenose dolphins were treated as the same species, *T. truncatus* (24)].

Two lists of the solitary and solitary-sociable delphinids and monodontids reported since 2008 are presented; one for Europe (where the majority of known solitary dolphins occur) and one for the rest of the world (see Tables 2, 3). Some of these animals are also included in the numbers in Table 1 as they were in residence in 2008 and continued to be so for a period afterwards (including, in some cases, up to the time of writing).

**Table 3:** Solitary and solitary-sociable dolphins in the rest of the world since 2008.

**Stage (Level) reached**	**Name of dolphin**	**Location**	Species^*^	**Gender**	**First seen**	**Last seen / date of death**	**References**
–	Q	Cape Chignecto, Nova Scotia, Canada	Dl	M	2008	Last sighted 19 August 2010 with serious injuries	(8); C. Kinsman 2018, pers. comm., 9 November; ^37^
–	Leucas / Luke / The Liverpool whale	Halifax, Nova Scotia, Canada	Dl	M	May 2015	Last sighted 9 August 2015 (apparently healthy)	C. Kinsman 2018, pers. comm., 9 November; ^38^^,^ ^39^
–	Nepisiguit Beluga	Bathurst, New Brunswick, Canada	Dl	M	June 2017	Last sighted in July 2018 accompanied by another beluga	^77^
5	Solitary Social Dolphin / Yera / Sally / Dolly / Beyoncé	New South Wales, Australia	Ta	F	Sept. 2012	No longer solitary	(9); ^40^^,^ ^41^
0	?	Coffs Harbor, New South Wales, Australia	Ta	?	?	Present	G. Storrie 2018, pers. comm., 1 May
4 (5)	Stinky/Humpy/Randy	Gran Cayman	Tt	M	Approximately 2009	2012	^42^^,^ ^43^^,^ ^44^
4 (4)	Pechocho	Gulf of California, Mexico	Tt	M	Approximately 1992	Present	(10); ^1^^,^ ^45^
4 (4)	Lucero	Veracruz, Mexico	Tt	F	Approximately 2003	Present	^46^
4 (4)^**^	Beggar / Mooch	Florida, USA	Tt	M	1990	Died 2012	(11); ^40^
4 (4)^**^	Dolphin 56	East Coast, USA	Tt	M	1979	Last seen Jul 2011	^47^^,^ ^48^
5	Kaimi	San Francisco, USA	Tt	?	Jul 2016	No longer solitary	W. Keener 2018, pers. comm., 23 February; ^49^
4 (5)	Jojo	Turks & Caicos Islands, West Indies	Tt	M	1980	Present	(2, 5); ^50^
4 (?)	Moko	North Island, New Zealand	Tt	M	March 2007	Died Jun 2010	I. Visser, 2018, pers. comm. 22 June; ^51^^,^ ^52^^,^ ^53^

* *Dl, Beluga (Delphinapterus leucas); Tt, Bottlenose dolphin (Tursiops truncatus); Ta, Indo-pacific bottlenose dolphin (Tursiops aduncus)*.

** *Interactions were limited to provisioning and touching*.

Since 2008, 36 solitary delphinoidea have been recorded including 27 bottlenose dolphins (25 *T. truncatus* and two *T. aduncus*), two striped dolphins (*Stenella coeruleoalba*), three common dolphins (*Delphinus delphis*) and four belugas (*Delphinapterus leucas*). Twenty-three of these animals were recorded in Europe and thirteen from other locations around the world. Of these, eleven are still solitary at the time of writing (“Dusty,” “Nimmo” and “Fungie” in Ireland, “Kylie” in Scotland, “Benny” in England, “Randy” and “Zafar” in France, “Pechocho” and “Lucero” in Mexico, “Jojo” in the Turks and Caicos Islands and the unnamed animal in Coffs Harbor, Australia).

Identifying individual solitary dolphins can be difficult if the animal does not have known distinctive markings and, also, if it is wide-ranging. In Ireland, for example, there appear, at first glance, to be quite a high number of solitary dolphins whereas actually, in some cases, the same dolphin has been given different names in different locations. For example, the dolphin known as “Dusty” in Doolin, County Clare, has now relocated to Inis Oírr where she is called “Sandy” (R. Meade, 2018, pers. comm., 7 May). She has also been referred to as “Mara” by some sources (^34^, R. Meade, 2018, pers. comm., 6 June). [“Mara” should not be confused with “Marra,” a solitary dolphin that was seen in Cumbria, UK and that died in 2006 (3)].

**Figure 1:**
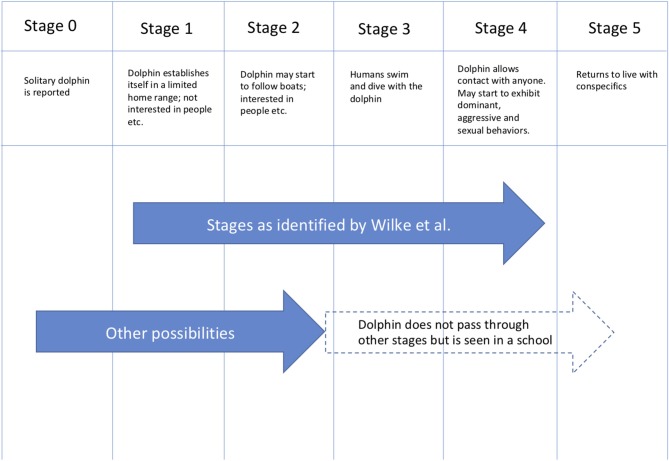
Solitary dolphin “sociability” stages [including some details adapted from Wilke et al. (2)].

We have attempted to categorize the dolphins (but not the belugas) according to the stages outlined in Figure 1 (see section Stages of Sociability for details). Categorizations are based on the information available to us in articles, personal communications and videos. We recognize that these may not include all interactions that have taken place between humans and a given dolphin and, therefore, may not fully represent the actual stage reached by each animal.

Although all of the dolphins listed in Tables 2, 3 exhibited solitary behavior, some of them did not, or have not yet, become “sociable” and are considered to be Stage 0 or Stage 1 in the sociability process. For example, the striped dolphin reported by Nimak-Wood et al. (19) was not observed in contact with humans although the authors considered that it did demonstrate behaviors which showed signs of related behavior, such as spending time close to a mooring buoy. One of the reasons put forward by the authors for the lack of human interaction is that the dolphin was present in a harbor where swimming was prohibited and therefore the opportunity for dolphin-human interaction was limited.

The short-beaked common dolphin described by Genov et al. (16), also did not exhibit the sociable behaviors and interactions with humans that characterize most solitary-sociable dolphins, but is included here because of its continued presence in one location over a period of time and because of its solitary nature. This dolphin could be considered to be Stage 1.

“Benny” the beluga is an interesting case because not only is it solitary, but it is also a long way from its native habitat. “Benny” was first sighted in the Thames Estuary in southeast England in July 2018 and was still present at the time of writing.^35^ Efforts are being made by the Port of London to keep the public away from the beluga, to restrict boat traffic in the area and to encourage people to observe the animal from a distance.^36^ So far, “Benny” has not exhibited any sociable behaviors.

Some dolphins are difficult to categorize because they do not seem to fit clearly into the stages and levels described. “Clet,” for example, as a great traveler, did not really reach Stage 3 because he did not allow certain humans to swim with him and so, perhaps, he should be considered Stage 2. However, he did demonstrate aggressive behavior toward people and other species (e.g., harbor porpoise) and directed sexual behavior toward the underside of a boat.^54^ Such behaviors would fit into Wilke's (2007) Level 5 which was proposed for animals in Stages 3 and 4, yet “Clet” did not appear to have reached either of these stages.

“Moko” in New Zealand, was clearly a Stage 4 dolphin but, it is not possible to allocate him to a particular level. He would only allow certain people to interact with him (Level 3) and yet he would sometimes demonstrate socio-sexual and dominance behaviors (Level 5) (I. Visser, 2018, pers. comm., 22 June). This demonstrates that, even though we can attempt to categorize and understand solitary dolphins, they are individuals and some animals may exhibit behaviors which do not appear to follow the pattern shown by the majority.

Of the 36 cases of solitary dolphins and belugas recorded since 2008, the sex of 25 of them has been recorded. Of these 25 animals, 18 (72%) of them have been identified as male and 7 (28%) were female. It is possible that mistakes were made in determining sex and so these figures do not necessarily show that males are more likely to become solitary than females.

### The Reassessment of the Stages of a Solitary-Sociable Dolphin

Based on a reassessment of available information from the last 10 years (as described above and in Tables 2, 3), we propose two additional stages; Stage 0 and Stage 5. A stage 0 dolphin is simply one seen to be persistently on its own; it is not seen within a limited home range and may be seen in multiple locations. It is distinguished from Wilke et al.'s Stage 1 animal that has established a home range.

Stage 5 relates to those seemingly rare cases where the dolphin returns to live with other conspecifics and ceases to be solitary. Such cases are hard to document because sometimes an individual disappears and it is not known whether it joined a pod, relocated or died. This Stage corresponds with the Stage 6 proposed by Goodwin and Dodds (5).

This proposed new categorization of stages (which incorporates those of Wilke et al. (2) is shown in Figure 1. The Figure also highlights that some individual dolphins do not necessarily pass through all stages. For example, a dolphin that is seen on its own for a period of time may subsequently join a school of other dolphins.

## Discussion—The Life Of The Solitary-Sociable Dolphin

In discussing the life of the solitary dolphin, we draw on examples not only from the last 10 years, but also some cases prior to 2008 where helpful.

### The Benefits of Interacting With Humans

**Figure 2:**
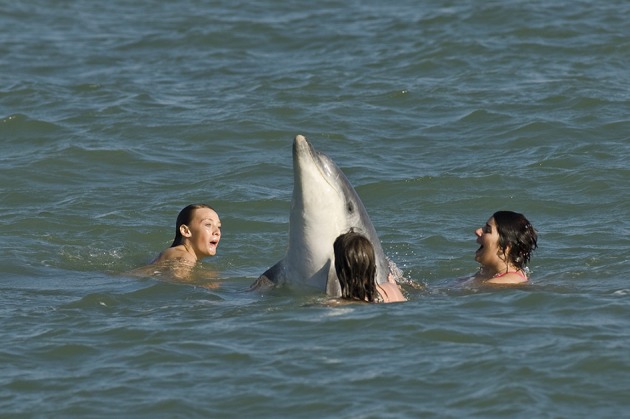
“Dave” the solitary-sociable dolphin, interacting with a group of people in Kent, UK (Photo: Terry Whittaker).

**Figure 3:**
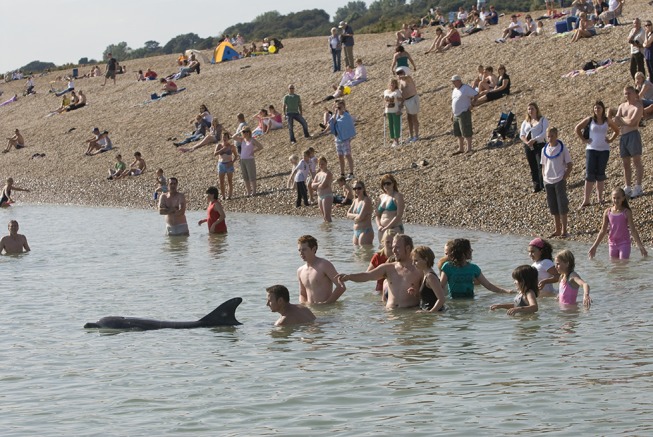
Large numbers of people traveled to see “Dave” and to interact with her (Photo: Terry Whittaker).

Solitary dolphins may seek out interactions with humans including touch, social, sexual and play behaviors (2). This contact, to some extent at least, seemingly replaces the social interaction and physical contact that would otherwise be provided by conspecifics (2) and can become very important to the individual animal. Bloom et al. (25) reported that a solitary dolphin in Northumberland, England regularly partook in interactions with swimmers and boats. The dolphin actively engaged in recreational activity with humans during 121 of 194 opportunities (62%) during the study period. Another dolphin living in British waters, “Dave” (see Figures 2, 3), was seen to spend almost a third of her time accompanied by humans or boats (26) whilst “Filippo,” the dolphin who lived in Manfredonia harbor in Italy, exhibited different behaviors within different areas of his home range. Within the port he was observed interacting with boats and humans only 16% of the time, whereas outside of the port he dedicated 65.9% of his time to this behavior (27).

Doak (23) suggested that the touch and social interaction provided by humans is important for the welfare of these solitary animals. In the case of “Pita,” a female bottlenose dolphin who was resident in Belize, she became calm and relaxed when swimmers swam slowly with her and gave her gentle rubs (28). Mizrahi et al. (29) reported that “Holly,” the Indo-Pacific bottlenose dolphin (*Tursiops aduncus*) resident off the Sinai Peninsula in Egypt, allowed people to touch her and then, later, sought out this physical contact. Although the dolphins may benefit, to a certain extent, from their interactions with humans, Lockyer and Müller (4) stated that the dolphins do not appear to depend on these interactions and that they are often temporary. For example, “Fanny” who regularly swam with a young girl, left her companion when she needed to find a new area to feed in and “Dolphy,” from Banyuls-sur-Mer in France, would only interact with people until her “dog companion” entered the water whereupon she would immediately ignore the humans (4).

### Welfare Concerns for Solitary-Sociable Dolphins

Concern for the welfare of free-ranging dolphins that interact on a regular basis with humans has been expressed by a number of authors (3, 22, 30). Human behavior can have a negative impact on the dolphin when the dolphin's needs are not taken into consideration and the dolphin is disturbed when it is resting or feeding (2). “Dave,” for example, did not feed when there were people in the water with her and her diving activity also altered, suggesting that she was foraging less (26). Such disturbances may be unintentional on the part of the humans but can have a negative impact on the welfare of the dolphin.

Unfortunately, people sometimes direct inappropriate behaviors toward the animals. Swimmers interacting with “Pita” were observed grabbing her fins, trying to ride her and touching sensitive areas, such as her genitals, face and blowhole (28). “Dave” was also subjected to these types of human behavior and parents were even seen putting their children on her back (26). Tourists interacting with wild botos in Brazil have also been observed trying to ride or restrain the dolphins, hitting them and feeding them inappropriate items (31).

Although food provisioning has rarely formed part of the development process for solitary-sociable dolphins, and some dolphins are reported to have refused the handouts offered to them (22), there are cases where solitary dolphins have been fed by humans. The solitary bottlenose dolphin known as “Beggar” was so named because he was regularly provisioned by humans and would beg for food by “orienting vertically in the water with his head out and mouth open…and accepting food,” (11). It is possible that “SC1” in Croatia and “Marra” in the UK were also provisioned by local fishing boats (18, 21). In Mexico, “Lucero” has been fed by local fishermen and tourists who come to swim with her (E. Morteo, 2018, pers. comm., 5 June). Such provisioning can become a welfare issue when dolphins are fed inappropriate or contaminated food items (11, 32) or, potentially, when they approach boats for food and are badly treated in return.

Provisioned dolphins also risk ingesting fish-hooks and other tackle. When “Beggar” was found dead in Florida, USA, it was concluded that he had been in poor health for some time and that this was partly attributable to his interaction with humans. He had wounds from boat strikes, fishing hooks and fishing line in his stomach as well as squid beaks which indicated that he had received more food from humans than from foraging on his own.^55^ Provisioning can also alter the dolphins' natural foraging habits (30). Studies reported by Foroughirad and Mann (33) have shown that provisioned female bottlenose dolphins living in the population in Shark Bay, Australia provided less care to their calves and that the calves had higher mortality rates than those born to non-provisioned females. After management measures were introduced to reduce the time that females spent being provisioned, calf survival rates increased (33).

Their close proximity to humans and presence in shallow waters puts solitary dolphins in danger of accidental stranding and injuries. “Marra,” for example, live stranded in May 2006 and it was suspected that this incident occurred because she was actively seeking human interaction and was spending time in shallow water (21). She was successfully refloated by British Divers Marine Life Rescue (BDMLR) and continued to interact with people until her death later that year.^56^

Wilke et al. (2) reported that anthropogenic causes have resulted in the deaths of a number of solitary dolphins; with proximate causes including oil spillages, underwater explosions, boat strikes and even death after being taken into captivity. Samuels et al. (22) reported solitary dolphins getting entangled in fishing gear and being wounded by fish hooks (including, in one case, in the eye).

**Figure 4:**
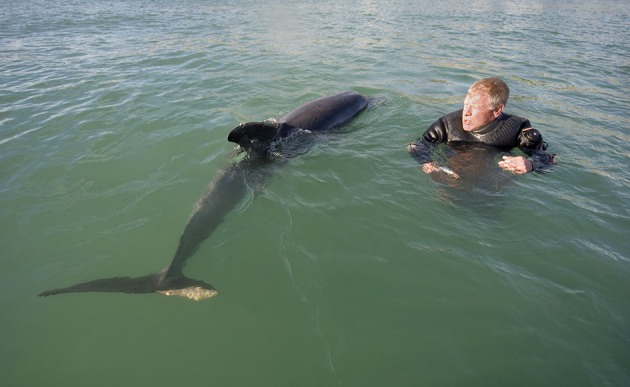
After “Dave” received a severe wound to her tail, attempts were made to give her fish laced with antibiotics (Photo: Terry Whittaker).

Bejder et al. (34) noted that when wild animals become habituated to contact with humans it can lead to negative outcomes for individual animals, for example if they lose their fear of motorized vehicles. In fact, many solitary-sociable dolphins have been injured by boats. “Freddie,” a solitary bottlenose dolphin in the north-east of England, was badly injured when he was struck by the propeller of a police launch (35), “Pita” had various scars which appeared to be caused by propellers (28) and the beluga known as “Q” was photographed with severe wounds on his back which may also have been the result of propeller strikes.^57^ The solitary striped dolphin reported in Croatia in 2008 had scars between its dorsal fin and tail which were possibly caused by a propeller (19) and “Dave” received a serious injury to her tail (probably caused by a propeller strike) which can be seen in Figure 4 (26). She disappeared shortly after receiving this injury and it is possible that she died from her wounds. “Marra” had various wounds in the months before her death possibly caused by a propeller and rope entanglement and she died from septicemia resulting from a bacterial infection which usually enters hosts through open wounds (21).

Elwen and Leeney (36) noted that some cetaceans may learn to avoid boats after a negative experience (such as a biopsy or capture) but most studies have shown that injuries do not lead to behavioral changes. Therefore, even if an animal has been struck by a boat, it will not necessarily learn to avoid boats in the future and is still at risk from further accidents. For example, the Heaviside's dolphin (*Cephalorhynchus heavisidii*) which Elwen and Leeney studied post-injury, continued to approach the research boat and to bow-ride (36).

Unfortunately, there are many reports of wild dolphins, including solitary-sociable dolphins, being deliberately harassed and injured (22, 32). This may be after they come into perceived or real conflict with people when their behavior disrupts human activity (such as fishing), damages property (for example, fishing gear) or when they exhibit aggressive behaviors (22). “Jojo” (who is still resident in Turks and Caicos) reportedly had 37 injuries between 1992 and 1999 all of which were related to interactions with people (22).

Deliberate attempts to shoot, spear or injure dolphins with a variety of weapons have been recorded (22, 32). For example, “Beaky,” who was seen in various places in the United Kingdom in the 1970s, had a scar from a healed bullet wound (4). Samuels et al. (22) reported that “Opo,” “Nudgy,” “Dobbie,” and “Costa Rican” (from New Zealand, USA, Israel, and Costa Rica, respectively) were all deliberately killed by humans. “Filippo” was also killed deliberately. He was found dead on 6th August 2004 having been stunned by an illegal fishing bomb and wounded with a harpoon (G. Pietroluongo, 2018, pers. comm., 2 April), The subsequent necropsy on his body found that it was riddled with shotgun pellets; evidence of earlier cruel interactions (G. Pietroluongo 2018, pers. comm., 2 April). Five other dolphins (“Percy,” “Tião,” “Horace,” “Simo,” and “Nina” from the UK, Brazil, New Zealand, Tunisia and Spain) may also have been killed as they disappeared mysteriously after negative interactions with local people (22).

### Interactions With Other Animals

Some solitary dolphins do interact, at least occasionally, with conspecifics. For example, “Holly” was occasionally seen with other dolphins and she even mated and gave birth to three calves during her “solitariness” (29). For the 4 years prior to her death in December 2004, “Holly” was accompanied by her only surviving female calf (29). “Pita” was also sometimes seen with members of her own species (28) and the presence of rake marks on “Percy” were taken to be evidence of interactions with other dolphins (37). “Beggar” was usually seen on his own but he was sometimes spotted with other dolphins some of whom copied his begging behavior (11). “Dolphin 56” could be seen with other dolphins on occasion but he demonstrated an interest in interacting with humans, that his conspecifics did not.^58^

“Françoise” was a bottlenose dolphin that belonged to a group which was resident in a lagoon on the Atlantic coast of France (4). She exhibited solitary-sociable behavior some of the time (approaching swimmers, divers and boats, bow-riding and rubbing against ropes) but she also spent time with her group when she actively avoided boats and swimmers. In Brittany, researchers saw “Fiete” with other bottlenose dolphins on two occasions. On the first of these occasions he prompted an unusually intense amount of socializing amongst the dolphins but also spent some of the time on his own at the back of the research vessel (M. Perri, 2018, pers. comm., 14 November). His interactions with other dolphins were alternated with periods of typical solitary dolphin behavior, such as spending hours following the same boat and swimming around a mooring buoy.

Sometimes solitary dolphins interact with individuals from other species. The striped dolphin studied in the Vinodol Channel in Croatia (“SC1”) was seen with another individual (species unknown) in 2006 and with a short-beaked common dolphin in 2008 (18). Similarly, the common dolphin known as “Kylie” has been seen interacting with a harbor porpoise and engaging in “affiliative” behaviors, such as travel, play and neutral association [(17); M. Cosentino, 2018, pers. comm., 12 March].

**Figure 5:**
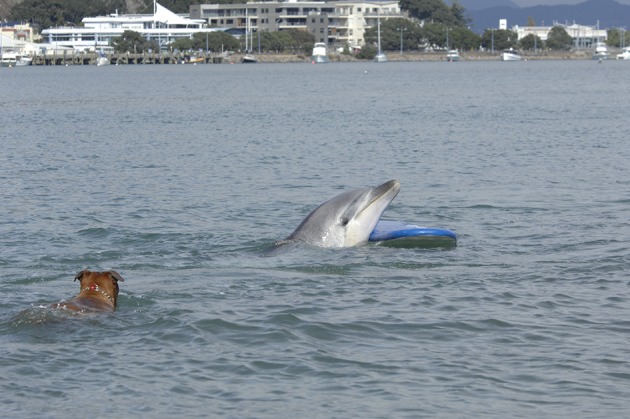
“Moko” with a surfboard he had “stolen” and a dog swimming after him (Photo: Ingrid Visser).

There are also cases of solitary dolphins interacting with domestic animals. “Dougie,” in Ireland, for example, regularly interacted with a pet dog who would swim with him every day^59^, “Dolphy,” “Beaky,” and “Simo” in Wales were known to have swum and played with dogs (4) and Figure 5 shows a dog approaching “Moko” in New Zealand.

### Risks to Human Safety

**Figure 6:**
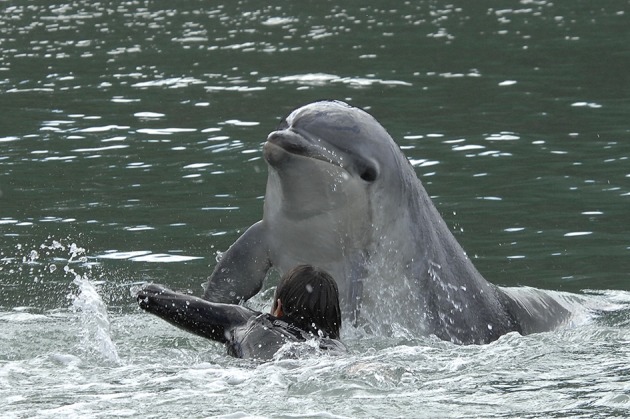
“Moko” being pursued by a swimmer in Whakatane, New Zealand (Photo: Ingrid Visser).

**Figure 7:**
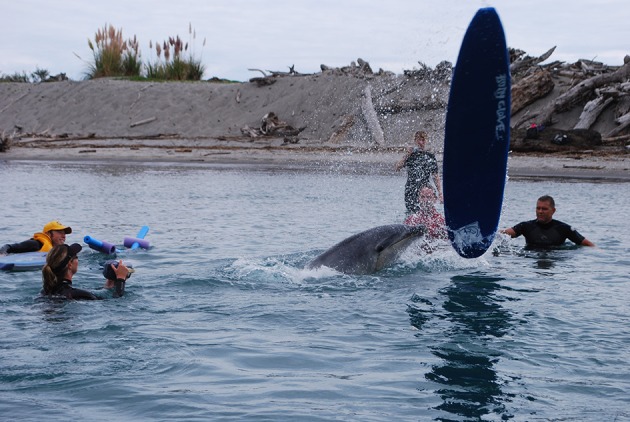
“Moko” playing with a surfboard by pushing it underwater to make it shoot up into the air (Photo: Ingrid Visser).

It is a commonly held belief that dolphins are of a friendly disposition and that they want to help and protect people who enter the water with them. Although there is evidence that this may sometimes be the case,^60^ there are also various accounts of solitary dolphins injuring humans in the water with them. Such aggressive behaviors from the dolphins are often the result of inappropriate human interactions (26, 38). Wilke et al. (2) suggested that inappropriate or overly energetic interactions on the part of humans may cause sexual arousal in the dolphin, which has the potential to turn into sexual aggression. In New Zealand “Moko,” for example, would exhibit socio-sexual and dominance behaviors if people who he was not interested in interacting with tried to make physical contact with him (I. Visser, 2018, pers. comm., 21 June). He would breach over people, push and bump them, knock them off surfboards and prevent swimmers returning to shore. Figures 6 and 7 show “Moko” interacting with people in the water

It has been suggested that male solitary-sociable dolphins are more likely to show aggressive behavior toward humans including sexually aggressive behavior (2, 9, 23). Samuels et al. (22) reviewed the behaviors of 29 solitary-sociable dolphins and stated that at least 13 of them had directed sexual behavior at humans, buoys and/or boats. It can be dangerous for humans to be on the receiving end of robust dolphin behavior (2). “Zafar,” who is currently resident in Brittany, has directed such attention to humans, pushing divers to the bottom and preventing kayaks from maneuvering.^61^

In the most extreme reported case of a violent interaction, “Tião,” a Brazilian bottlenose dolphin, injured 29 people and caused the death of a 30-year-old man after butting him; the man died from internal injuries (38). The context of this may be important. People had subjected “Tião” to a number of inappropriate behaviors such as grabbing his fins, hitting him, jumping on him and even trying to insert ice-lolly sticks into his blowhole (38) which would have been potentially life-threatening as it could have impaired the dolphin's ability to breathe and dive. There is another case, from Gran Canaria, Spain in 2001, where robust interactions with a free-swimming dolphin appear to have resulted in the death of a swimmer (39).

Various videos uploaded to YouTube show “Dusty” the dolphin exhibiting aggressive behavior toward humans who try to approach her in the water ^62^^,^ ^63^ and Berrow (40) reported that she damaged one woman's ribs by ramming her, whilst another swimmer suffered internal hemorrhaging. Samuels et al. (22) reported that some solitary-sociable dolphins have “abducted” people who later needed to be rescued by boat. “Percy” was reported to have pushed a swimmer out to sea in south-west England and “Dave,” “Marra,” and “Georges” (all dolphins seen in UK waters) were often “rough” with their human “playmates” and stopped people from leaving the sea on occasion (3, 4, 21). “Pita” was also aggressive toward people when they tried to leave the water and would push and bump into swimmers and occasionally rub her genitals against them (28). “Beaky” would butt people aggressively, usually when there were a lot of people in the water with him (4). “Dave” and “Marra” were both seen breaching on top of a number of people, which could have caused serious injury (21, 26). In the UK, there was also concern for the safety of the people who swam with “Georges” including risks not directly related to the dolphin, such as people swimming out of their depth or risking hypothermia (21).

Humans who interact with dolphins may also be at risk of being bitten. “Beggar,” the dolphin who was regularly fed by humans, was reported to have bitten people on various occasions and this sometimes resulted in a need for medical treatment (11). “Percy” also reportedly bit people when he was “over-excited” due to a large number of people being in the water with him at the same time or when he felt that his playtime with a known human was threatened (4, 37). In Brazil, provisioned wild botos have also bitten humans (31).

The risks to human health and safety as well as the welfare concerns apparent for the dolphins themselves, highlight the need for specific management and protection for these animals.

### Protection for Solitary-Sociable Dolphins

Wilke et al. (2) reported that there did not appear to be specific legislation in place protecting solitary-sociable dolphins anywhere where these animals were present, although many countries have regulations for watching or swimming with cetaceans in general.

The widespread human desire to approach or interact with dolphins inappropriately, in ways which can be dangerous to both dolphins and people, means that the management of human behavior is necessary (3, 7). Unfortunately, this sometimes comes too late and in Brazil, in 1994, it was only after “Tião” had fatally injured someone that a management plan was put into place (38). The plan involved educating the public, working with the media and trying to prevent dangerous interactions between the public and the dolphin (38). No further accidents or incidents were recorded before the dolphin apparently left the area, proving that the management plan protected the humans interacting with the dolphin although it is unclear whether it protected the dolphin, as it is possible that “Tião” was deliberately killed by people taking “revenge” for his role in the death of a human (22).

**Figure 8:**
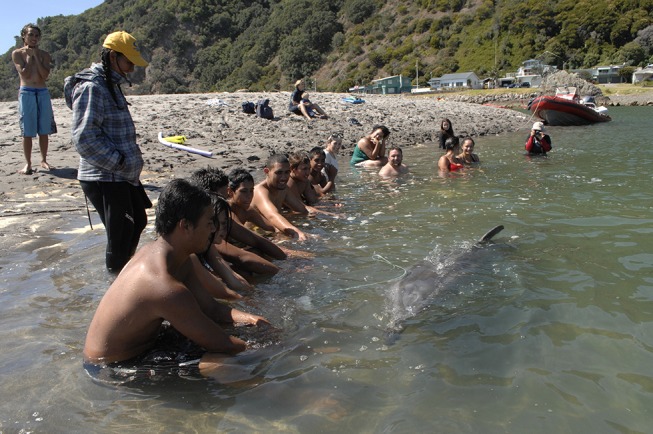
“Moko” the solitary-sociable dolphin interacting with the public under the supervision of his guardian (wearing the yellow cap) (Photo: Ingrid Visser).

Some efforts to manage interactions between humans and dolphins have improved the survival chances for certain solitary-sociable dolphins (22). In Ireland, the Irish Whale and Dolphin Group (IWDG) has held various public meetings to address management issues regarding solitary dolphins (40). Doak (23) reported that some communities inform the public, via notice-boards and leaflets, about best practice when it comes to how to treat the local solitary dolphin. In some cases, special “guardians” have been assigned to ensure that the dolphin is safe and not harassed or injured by the public (23). In France, the dolphins “Dolphy” and “Fanny” both had guardians who worked alongside local supporter groups and under the supervision of the University of Marseille (23). Some of “Moko”'s interactions with people were monitored by a guardian (see Figure 8).

It is important that the guardians are working to protect the dolphins and to inform the public. Some solitary dolphins have developed relationships with specific humans who interact with them regularly, for example “Mara” (“Dusty”/“Sandy”) in Ireland and “Jojo” in the Turks and Caicos.^64^^,^ ^65^ According to Wilke et al. (2), humans who form relationships with solitary dolphins can become possessive regarding “their” dolphin and this may lead to problems for the dolphin if the person is not inclined to share information about what the dolphin does and does not like regarding interactions with humans.

When “Dave” was resident in southeast England she attracted a lot of attention (see Figure 3) and local volunteers worked with non-governmental organizations to patrol the beaches, monitor the dolphin, talk to members of the public and put up posters about the potential dangers for both swimmers and dolphin during interactions (26). Such was the public's enthusiasm to get into the water with the dolphin that the local police were also called in on several occasions to help protect her and two arrests were made. The Kent Tourist Board, local council and other agencies were involved in meetings about dolphin and human safety (26).

In New Zealand, local companies that took people to swim with dolphins, actively helped protect a solitary-sociable dolphin called “Maui” by limiting the number of people allowed in the water with her, or by not taking tourists to swim with her at all (41). As the Department of Conservation also strictly enforced regulations, “Maui” had limited contact with people. In the Turks and Caicos, “JoJo” has been officially protected since the 1990s.^66^ The “JoJo Dolphin Project” promotes legal protection for marine mammals (including “JoJo”), and aims to educate the public on how to interact with wild dolphins.^67^

In Belize, human interactions with “Pita” (which could include her being approached by up to four boats at a time or having as many as 30 people in the water with her) were, generally, not supervised although some guidelines about dolphin-human interactions were available (28). In Brazil, activities involving interactions with wild dolphins, such as feeding and swimming with botos is not regulated, codes of conduct are often not followed and those running businesses promoting these interactions do not receive any specialized training (31). In general, there is a lack of legislation relating to tourism involving animals in Brazil and that regarding dolphins is limited to preventing disturbance by boats (31). Alves et al. (31) recommended the development of specific legislation to regulate feeding, touching and swimming with botos. The Ministério Públic Federal (Public Prosecutor's Office) has recently released a recommendation that tourist operators in the Amazonas should stop promoting physical interactions between tourists and wild animals.^68^

In some countries, conservation legislation may offer some protection to solitary-sociable dolphins. In the United Kingdom, dolphins are protected by the Conservation of Offshore Marine Habitats and Species Regulations 2017, the Conservation of Habitats and Species Regulations 2017 and the Wildlife and Countryside Act 1981 (as amended).^69^ Both of the 2017 Regulations state that “a person who deliberately disturbs wild animals…is guilty of an offense,” (42, 43). Disturbance includes that which “is likely to impair their ability…to survive, to breed or reproduce, or to rear or nurture their young,” (42, 43). Part 9 of the Wildlife and Countryside Act 1981 states that “if any person intentionally or recklessly disturbs any wild animal included in Schedule 5 as (a) a dolphin or whale (cetacea), … he shall be guilty of an offense” (44). As interactions with solitary-sociable dolphins have been shown to lead to injuries which could impair their survival prospects as well as to incidents which have led directly to the deaths of various dolphins, it is not unreasonable to refer to interactions with these animals as “disturbances” and, therefore, offenses according to these laws.

In both the USA and New Zealand, it is illegal to “harass” dolphins (45, 46). In New Zealand, “disturbing” a dolphin is also considered an offense under The New Zealand Marine Mammal Protection Act 1978 (46). The New Zealand Marine Mammals Protection Regulations (47) state that “persons may swim with dolphins…but not with juvenile dolphins.” Details about how vessels should behave around dolphins is given in detail. The Australian National Guidelines for Whale and Dolphin Watching 2017 also provide detailed information regarding which types of boats can partake in dolphin watching and how they can and cannot approach the animals with details about caution zones and no-approach zones (48). Regarding “habituated solitary dolphins” the Guidelines state that feeding, touching or swimming with these animals is “not beneficial to the dolphin and puts the animal at greater risk of injury or death due to boat strike or entanglement. In addition these interactions are often in breach of state regulations,” (48).

Many countries discourage the public from swimming with dolphins. In Ireland the National Parks and Wildlife Service (NPWS) produced guidelines in 2000 reminding people that dolphins are wild animals and discouraging interactions, such as swimming with them or attempting to manhandle or interfere with them (40). The Department of Transport, Tourism and Sport (49) published guidelines for interactions with whales and dolphins in Irish coastal waters including details of how vessels should be handled around cetaceans and stating that people should not attempt to swim with them. Swimming with dolphins is not recommended in Australia unless the relevant authority has given permission and it is recommended that “if approached by a whale or dolphin a swimmer must move slowly to avoid startling the animal and must not attempt to touch it or swim toward it,” (48). “Attempts at swimming with whales or dolphins should stop if the animals show signs of disturbance or agitation,” (48).

Sometimes it may be necessary for the authorities to specifically prohibit certain interactions. In France, the mayor of Penmarc'h, Brittany, put an order in place to prevent people from swimming with “Zafar” in 2017.^70^ The Cayman Islands Government advises the public not to swim with wild dolphins, noting that lone dolphins in particular can be unpredictable and dangerous.^71^ In the USA, NOAA Fisheries (2018) state that the viewing of marine mammals must be carried out in a way that does not harass the animals and closely approaching or interacting (or attempting to interact) with whales, dolphins and porpoises is discouraged including swimming with them, petting them, touching them or trying to get a reaction from them.^72^

In some places there are schemes in place to encourage responsible encounters with cetaceans. The Dolphin SMART program in the USA aims to promote responsible dolphin watching practices (50). It encourages people to;

“Stay at least 50 yards from dolphins,

Move away cautiously if dolphins show signs of disturbance,

Always put your engine in neutral when dolphins are near,

Refrain from feeding, touching, or swimming with wild dolphins,

Teach others to be Dolphin SMART.”

The UK's WiSe Scheme trains vessel operators on how to best approach and interact with marine wildlife whilst minimizing disturbance.^73^ As well as their cetacean code of conduct, they have developed a further “Sociable, solitary dolphin code of conduct” which highlights the fact that maintaining a distance from these animals can be impossible if the animal approaches the vessel (WiSe Scheme, 2018)^73^. The Code of Conduct says that it is important to ensure that solitary-sociable dolphins do not follow vessels into harbors/marinas but that, if they do, the harbor authorities must be informed and that they should contact BDMLR or Marine Connection to find out whether further action needs to be taken.

The WiSe Scheme (2018) highlights the risk of injury from propellers and the fact that solitary-sociable dolphins are often very attracted to them and therefore it is recommended that engines are put into neutral if a dolphin approaches. Boat owners are also warned of the danger posed to passengers if a dolphin rubs against the boat, rudder or propeller, subsequently unsteadying the vessel. If the dolphin does not move away, even after turning off the engine, it is recommended to return to harbor with a steady speed. Feeding and resting areas should be avoided and if the animal is seen engaging in these behaviors it should not be approached. For boat owners operating in an area with a resident solitary-sociable dolphin, it may be appropriate to fit a propeller guard. It is important not to swim with, touch or feed these dolphins as such behavior can lead to the animal being injured or disturbed and even to its death.

Simmonds (3) called for better protection for solitary-sociable dolphins and for increased efforts to prevent them from becoming accustomed to contact with humans in the first place. Hawkins (9) proposed a number of ways, including relocation, in which the habituation of a solitary dolphin could be avoided. Solitary dolphins which do not become accustomed to interacting with humans may have an increased chance of starting to interact with conspecifics again. “Kaimi,” for example, was solitary for 1 year in San Francisco Bay, USA before being joined by an adult female (possibly her mother) (W. Keener, 2018, pers. comm., 4 June). Recently, they have been joined by two further dolphins (W. Keener, 2018, pers. comm., 4 June). In terms of the Stages (see Figure 1), “Kaimi” went from Stage 0 to Stage 5 without passing through the other stages.

Santos (38) also recommended that regulations and guidelines are needed to prevent dolphins and humans from being injured. Such guidelines may need to be specific to certain circumstances. In tropical countries, for example, people are in the water almost all year round and all day long. When “Tião” was in Brazil, he often had as many as 300 people in the water with him (M. Santos, 2018, pers. comm., 19 March).

A management plan can help protect solitary-sociable dolphins and, according to Hawkins (9), should include “(1) stakeholder engagement, (2) monitoring and research, (3) management responses, compliance and enforcement, (4) communication strategy, (5) public education and (6) environmental considerations.”

Wilke et al. (2) recommended that those who manage solitary-sociable dolphins should consider whether it is possible for the dolphin to return to normal dolphin society and to assist in this outcome if possible. A solitary-sociable dolphin from Australia, which had progressed to stage 4 of sociability, was relocated in 2013 and later joined a group of wild dolphins (9);^74^. This dolphin was young (between 3.5 and 4.5 years old) when first sighted and its solitary-social period was relatively short-lived. After about 4 months of being solitary and isolated in St George's Basin, the dolphin would allow physical contact from swimmers. After detailed assessment it was decided to relocate the dolphin to the open ocean.^75^ Although she continued to interact with people for a time, these interactions gradually decreased and she appeared to reintegrate into dolphin society 9; ^76^.

Another translocation took place in 2017, of a solitary male beluga who was moved from New Brunswick to the St Lawrence Estuary in Canada.^77^ He was not yet exhibiting sociable behavior and it was considered appropriate to move him to an area where he could find other belugas and contribute to the breeding population there. A year later, in July 2018, he was sighted in the company of another male beluga off Cape Breton in Nova Scotia. This sighting was not in an area inhabited by his natal or any other beluga population and it is not clear at the time of writing whether he has returned to live with conspecifics or not (C. Kinsman, 2018, pers. comm., 9 November).

As well as managing human behavior around solitary dolphins, in some cases, it may be possible to “train” the dolphin so that certain behaviors are discouraged (9). According to Wilke et al. (2) there was some short-term success at teaching “Jojo” not to sexually display toward humans and teaching dolphins to re-socialize with conspecifics might be a possibility in some cases.

## Recommendations For Management Of Solitary-Sociable Dolphins

It is clear that solitary dolphins are particularly vulnerable and that their interactions with humans need to be carefully managed to prevent them from being negatively impacted (51). Such careful management can also ensure that humans are not injured or put at risk either. How a specific dolphin should be managed will depend on its sex, age and personality (2). The size and character of the dolphin's home range will also influence what kind of management is needed and what is feasible (2).

Although it may appear that discouraging interactions between humans and dolphins is in the dolphin's best interest, it has been suggested that a dolphin in the later stages of sociable-solitariness may receive some benefit from its interaction with humans in terms of improved welfare (2). We recommend that the stage of sociability needs to be considered when a management plan is being devised. For example, dolphins in stages 0, 1 and 2 may be best protected by strictly discouraging and limiting interaction with them. For dolphins in later stages it may be argued that the situation is different but if, for whatever reasons, human interactions are permitted they clearly require very strict supervision to try to ensure that the dolphins are not disturbed or injured and, likewise, that human health and safety is guaranteed. The sea should always be viewed as a dangerous environment and people entering the water to interact with large wild predators need to be fully cognizant of the risks.

Appropriate protection will need to be implemented by government officials who can limit access to the dolphin and who can ensure that a management plan is developed for the specific situation and adapted as necessary depending on how things change over time (2). Local stakeholders, such as fishermen will need to be involved and people from the local community can also be recruited to help educate the public. Guidelines about how to interact with the dolphin are essential including the basic tenet that observation is preferable to interaction. This will also ensure that people are not at risk of injury or other negative consequences from interacting with the dolphin.

Wilke et al. (2) recommended various points that could be included in a management plan:

- An off-limits area where humans are not allowed to enter thus allowing the dolphin to feed or rest without being disturbed;
- A limit to how many people interact with the dolphin at any one time;
- Restricting the number and/or type of boats which can approach the dolphin, particularly considering the risk of propeller injury;
- Promoting good behavior and respect between boat owners so that no conflict arises between those trying to approach the dolphin;
- No touching of the dolphin's sensitive spots (blowhole, eyes, genital area), and,
- No feeding of the dolphin.

The importance of using diplomacy and good communication skills at all points is essential (2). If conflict arises between those people who want to interact with the dolphin, or if people who use the area where the dolphin lives believe that their needs are not being taken into consideration, resentment can grow which can have negative consequences for the dolphin and the humans involved in the conflict.

We also propose that to adequately protect solitary-sociable dolphins through the implementation of a management plan, it is necessary to clearly define what constitutes disturbance and harassment, so that it is clear which human behaviors are acceptable and which are not.

## Conclusion

The fates of many solitary-sociable dolphins show that these animals are, to a great extent, at the mercy of people's desire to interact with them. Their welfare can be negatively impacted if they are disturbed whilst they attempt to rest or forage, are insensitively touched and prodded by those who wish to commune with them, are fed inappropriate items or are accidentally struck by boats. There are also cases of dolphins being deliberately injured or killed by those who take a dislike to them or who seek some kind of “revenge.”

Depending on where these animals live they may receive some form of protection under the law or due to the diligence of local people but there exist no general guidelines as to how they should be managed during the different stages of sociability. We recommend above (section Recommendations for Management of Solitary-Sociable Dolphins) how solitary and solitary-sociable dolphins can be better protected.

In summary, Stage 0 dolphins should be monitored but left alone unless they get into trouble whilst Stages 1–4 dolphins need to be monitored and a management plan developed for their safety and that of the people who come into contact or choose to interact with them. The following mnemonic may help to get the message across about how to protect solitary dolphins:

Dolphin CARE:

Choose not to disturb or otherwise interact with the dolphin

Alert the authorities if necessary

Respect the dolphin

Enjoy watching from a distance.

## Author Contributions

LN researched the current cases of solitary-sociable dolphins and wrote most of the manuscript. MS proposed the topic and supervised, edited, and contributed various sections.

### Conflict of Interest Statement

The authors declare that the research was conducted in the absence of any commercial or financial relationships that could be construed as a potential conflict of interest.

## Footnotes

^1^https://riodoce.mx/sincategoria/el-pechocho-delfin-silvestre-domesticado

^2^https://www.mwdw.net/clet-the-dolphin-seen-in-manx-waters/

^3^http://www.al-lark.org/2017/03/fiete-a-great-traveler-dophin.html

^4^http://www.seawatchfoundation.org.uk/unusual-solitary-dolphin-moves-to-aberystwyth/

^5^https://www.facebook.com/clydeporpoise/posts/1716608158447857

^6^http://www.bdmlr.org.uk/index.php?mact=News,cntnt01,detail,0&cntnt01articleid=1012&cntnt01origid=15&cntnt01returnid=54

^7^https://www.kentonline.co.uk/gravesend/news/benny-could-be-here-to-stay-192434/

^8^http://www.spiegel.de/international/zeitgeist/rudolf-the-bottlenose-dolphin-frolics-on-belgian-coast-a-711454.html

^9^https://www.croatiaweek.com/swimmers-warned-not-to-swim-with-bobi-the-bottlenose-dolphin/

^10^https://www.blue-world.org/bobi-solitary-adriatic-dolphin/

^11^https://www.youtube.com/watch?v=L0YBiIFsqTM

^12^https://www.24sata.hr/fun/kupace-u-slanom-iznenadio-dupin-koji-se-brckao-s-njima-528246

^13^http://www.iwdg.ie/news/?id=2572

^14^https://www.independent.ie/irish-news/watch-dusty-the-wild-atlantic-way-dolphin-gives-fungi-a-run-for-his-money-with-show-for-island-daytrippers-35888275.html

^15^https://www.irishtimes.com/news/ireland/irish-news/second-dolphin-takes-up-residence-off-aran-island-1.1939115

^16^http://www.irishdolphins.com/webpilot/list/details.asp?l=10&contentid=57

^17^http://connachttribune.ie/galways-latest-maritime-attraction-performs-crowds-898/

^18^https://www.irishcentral.com/news/amazing-footage-of-a-dog-playing-with-a-dolphin-off-the-coast-of-ireland-video-127888298-237406421

^19^https://video.nationalgeographic.com/wild/unlikely-animal-friends/00000144-16d7-dcf1-a954-57df5eb90000

^20^https://www.independent.ie/irish-news/news/dont-panic-beloved-fungie-the-dolphin-suffers-deep-gash-from-boat-propeller-34769753.html

^21^http://www.thejournal.ie/dolphin-solitary-cork-1502921-Jun2014/

^22^http://www.marine-life.org.uk/2015-articles/clet,-the-cosmopolitan-dolphin-%28160315%29

^23^http://www.kn-online.de/Kiel/Frankreich-Delfin-Fiete-macht-Urlaub-in-Saint-Malo

^24^http://www.laopinioncoruna.es/coruna/2009/07/16/delfin-gaspar-asusta-buzos-puerto-fontan/304305.html

^25^http://www.abc.es/20101213/local-galicia/delfin-gaspar-201012130911.html

^26^https://www.youtube.com/watch?v=O2hgKXDt3eg

^27^https://voilesetvoiliers.ouest-france.fr/croisiere/grande/voilier-loick/un-dauphin-pas-commun-24ef3743-1cf2-7148-8fa7-24ff3fb9f7cd

^28^https://www.reseaucetaces.fr/groupe-de-travail-sur-les-dauphins-solitaires-et-familiers/dauphins-solitaires-and-familiers-en-france/

^29^http://www.bdmlr.org.uk/index.php?mact=News,cntnt01,detail,0&cntnt01articleid=991&cntnt01pagelimit=15&cntnt01returnid=54

^30^https://www.facebook.com/Randy-Dony-le-dauphin-1672284889658475/

^31^http://maisouestelcano.blogspot.com

^32^https://www.youtube.com/watch?v=onl3GFlDzj8

^33^https://vimeo.com/223415504

^34^http://www.dailymail.co.uk/news/article-2024070/Meet-real-life-Flipper-The-extraordinary-relationship-woman-wild-dolphin-friend.html

^35^http://www.bdmlr.org.uk/index.php?mact=News,cntnt01,detail,0&cntnt01articleid=1012&cntnt01pagelimit=15&cntnt01returnid=54

^36^https://www.pla.co.uk/BDMLR-and-PLA-joint-press-statement

^37^https://www.ctvnews.ca/q-a-lost-beluga-whale-thrills-maritimers-1.315665

^38^https://www.cbc.ca/news/canada/nova-scotia/beluga-whale-sighting-in-liverpool-harbour-worries-marine-biologist-1.3119905

^39^http://www.whalestewardship.org/www.whalestewardship.org/Leucas_2.html

^40^http://www.balmoralbeachclub.com.au/storytellers-the-balmoral-dolphin/

^41^https://www.theguardian.com/environment/2014/jan/07/lonely-dolphin-making-human-friends-but-experts-ask-swimmers-to-stay-away

^42^https://www.caymancompass.com/2012/10/01/marine-experts-to-examine-lone-dolphin/

^43^http://www.foxnews.com/science/2012/10/11/us-scientists-puzzled-by-lone-dolphin-in-cayman-islands-deemed-dangerous.html

^44^https://www.caymancompass.com/2013/01/07/top-stories-of-2012-lone-dolphin-becomes-fixture-in-cayman-waters/

^45^https://www.facebook.com/periodicoeldebate/posts/10156096436855903

^46^http://www.eluniversal.com.mx/estados/lucero-la-delfin-que-decidio-no-volver-al-mar#imagen-1

^47^https://www.outsideonline.com/1825461/dolphin-56

^48^http://www.nj.com/news/index.ssf/2012/08/dolphin_56_where_are_you.html

^49^https://www.sfgate.com/science/article/Warmer-waters-have-more-bottlenose-dolphins-10831036.php#item-85307-tbla-4

^50^http://turksandcaicostourism.com/turks-caicos-attractions/jojo-the-dolphin/

^51^http://www.projectjonah.org.nz/Teacher+Resources/Solitary+Dolphins.html

^52^http://www.projectjonah.org.nz/Teacher+Resources/Solitary+Dolphins/Moko+the+dolphin.html

^53^https://www.nzherald.co.nz/nz/news/article.cfm?c_id=1&objectid=10657370

^54^http://www.seawatchfoundation.org.uk/clet-the-travelling-bottlenose-dolphin/

^55^https://start1.org/news/beggar-the-dolphin-did-contact-with-humans-lead-to-his-death/

^56^https://www.seawatchfoundation.org.uk/bottlenose-dolphin-trapped-in-maryport-harbour-rescued-and-set-free/

^57^http://www.whalestewardship.org/www.whalestewardship.org/Q.html

^58^https://www.youtube.com/watch?v=Icq0CHQV3js

^59^https://www.youtube.com/watch?v=l2vU8U0j_4E

^60^https://www.dolphins-world.com/dolphins-rescuing-humans/

^61^http://zafarledauphin.blogspot.com

^62^https://www.youtube.com/watch?v=-X09Y1aEtM0

^63^https://www.youtube.com/watch?v=B3RIpd0FiE4)

^64^https://www.youtube.com/watch?v=jM6WTXSmG78

^65^http://deanandjojo.org/dean-and-jojo_wild-dolphin/

^66^https://www.reseaucetaces.fr/groupe-de-travail-sur-les-dauphins-solitaires-et-familiers/dauphins-ambassadeurs-daujourdhui/

^67^http://www.ieyenews.com/wordpress/the-jojo-dolphin-project/

^68^http://www.mpf.mp.br/am/sala-de-imprensa/docs/recomendacao-ecoturismo-e-interacao-animais-silvestres

^69^https://www.gov.uk/government/publications/protected-marine-species/cetaceans-dolphins-porpoises-and-whales

^70^https://www.letelegramme.fr/finistere/quimper/penmarc-h-interdit-de-nager-pres-des-dauphins-04-11-2017-11728087.php

^71^http://doe.ky/marine/whales-and-dolphins/

^72^https://www.fisheries.noaa.gov/topic/marine-life-viewing-guidelines

^73^https://www.wisescheme.org

^74^https://www.dolphinresearchaustralia.org/dolphin-rescue-sussex-inlet/

^75^http://www.environment.nsw.gov.au/media/OEHMedia13050201.htm

^76^http://whalespotter.com.au/?p=1815).

^77^https://gremm.org/en/le-beluga-de-la-riviere-nepisiguit-est-revu-bien-vivant/
