# Corrigendum: A Global Reassessment of Solitary-Sociable Dolphins

**DOI:** 10.3389/fvets.2019.00212

**Published:** 2019-07-02

**Authors:** Laetitia Nunny, Mark P. Simmonds

**Affiliations:** ^1^Wild Animal Welfare, Barcelona, Spain; ^2^School of Veterinary Science, University of Bristol, Bristol, United Kingdom; ^3^Humane Society International, London, United Kingdom

**Keywords:** bottlenose dolphin, solitary dolphin, sociable dolphin, lone dolphin, solitary-sociable dolphin, beluga, animal welfare

In the original article, there were mistakes in Table 2 and Table 3 as published. The references for the dolphin “Doogie/Dougie” in Table 2 and those for the dolphins “Q, Leucas/Luke/The Liverpool whale, Solitary Social Dolphin/Yera/Sally/Dolly/Beyoncé, Stinky/Humpy/Randy, Pechocho, Lucero, Beggar/Mooch, Dolphin 56, Kaimi, Jojo and Moko” in Table 3, were incorrectly published.

**Table 2:** Solitary and solitary-sociable dolphins in Europe since 2008.

**Stage (Level) reached**	**Name of dolphin**	**Location**	Species^*^	**Gender**	**First seen**	**Last seen / date of death**	**References**
1	Unnamed	Monfalcone, Italy	Dd	Presumed F	Jun. 2010	Aug. 2011	(16)
1	Stormy	Wales, UK	Dd	?	Dec. 2014	Apr. 2015	^4^
3 or 4 (2 or 3)	Kylie / Colin / Donna	Firth of Clyde, Scotland, UK	Dd	F	Approximately 2001	Present	(17); M. Cosentino, 2018, pers. comm., 2 June.; ^5^
–	Benny	Thames Estuary, UK	Dl	?	July 2018	Present	^6, 7^
1 or 2	SC1	Vinodol Channel, Croatia	Sc	F	Aug. 2004	Last seen Jul. 2009	(18)
1	?	Mali Lošinj harbor, Croatia	Sc	?	Aug. 2008	Last seen 11 Sept. 2008	(19)
1 or 2	Rudolf	Ostend & Nieuwpoort, Belgium	Tt	?	2007	Last seen 2008 (or possibly 2010)	(20); ^8^
2	? (possibly Rudolf)	Knokke-Heist, Belgium	Tt	?	Jul. 2010	Last seen Oct. 2010	(20)
4 (4)	Bobi	Karin Sea, Croatia	Tt	M	Apr. 2014	Last seen 2016 (or possibly 2017)	^9, 10, 11, 12^
2	? (possibly Bobi)	Slano, Croatia	Tt	?	Jun. 2017	Seen a few times. Possibly still present	D. Crljen, 2018, pers. comm. 18 June;^12^
4 (5)	Dusty / Sandy / Mara	North Clare / Inis Oírr, Ireland	Tt	F	2000	Present	R. Meade, 2018, pers. comm. 7 May; ^13, 14, 15, 16, 34, 64^
1	Nimmo / Salty	Galway, Ireland	Tt	?	Since at least 2008	Present	^17^
2	Doogie / Dougie	Tory Island, Ireland	Tt	F	2006	Last seen? 2008?	(5); ^18, 19, 64^
4 (3)	Fungie	Dingle, Ireland	Tt	M	1983	Present	^20^
2 (5)	Clet / Nick / Nobby / George II / Hobnob	France, UK, Ireland	Tt	M	2008	Last seen summer 2015	^2, 21, 22, 54^
1	Fiete / Freddy	Brittany, France and Kiel, Germany	Tt	M	2016	Aug. 2017	M. Perri, 2018, pers. comm., 18 May; ^3, 23^
2	Gaspar / Jean Copo'h / Jean Floc'h	Brittany, France and Galicia, Spain	Tt	M	2003	Last seen 2010	^24, 25, 26^
1	Lilou / Wifi	Brittany, France	Tt	M	2007	?	^27, 28^
4	Randy / Dony / Georges	UK, Ireland, France, Holland, Belgium.	Tt	M	April 2001	Present (Currently in Brittany, France)	^29, 30^
3 (3 or 4)	Elcano	Northern Spain, Western France	Tt	M	Feb. 2013	Last seen Sept. 2013	^31, 32^
3 or 4 (5)	Zafar / Toto	Brittany, France	Tt	?	Jun. 2017	Present	^61, 33^
0	?	Portsmouth & Isle of Wight, UK	Tt	?	Jun. 2017	?	^29^
3 or 4 (2)	Splashy	Cornwall, UK	Tt	M	Jul. 2017	Last seen Mar. 2018	D. Jarvis 2018, pers. comm., 29 May; A. Lowe 2018, pers. comm., 16 March

* *Dd, Short-beaked common dolphin (Delphinus delphis); Dl, Beluga (Delphinapterus leucas); Sc, Striped dolphin (Stenella coeruleoalba); Tt, Bottlenose dolphin (Tursiops truncatus); Ta, Indo-Pacific bottlenose dolphin (Tursiops aduncus)*.

**Table 3:** Solitary and solitary-sociable dolphins in the rest of the world since 2008.

**Stage (Level) reached**	**Name of dolphin**	**Location**	Species^*^	**Gender**	**First seen**	**Last seen / date of death**	**References**
–	Q	Cape Chignecto, Nova Scotia, Canada	Dl	M	2008	Last sighted 19 August 2010 with serious injuries	(8); C. Kinsman 2018, pers. comm., 9 November; ^37^
–	Leucas / Luke / The Liverpool whale	Halifax, Nova Scotia, Canada	Dl	M	May 2015	Last sighted 9 August 2015 (apparently healthy)	C. Kinsman 2018, pers. comm., 9 November; ^38, 39^
–	Nepisiguit Beluga	Bathurst, New Brunswick, Canada	Dl	M	June 2017	Last sighted in July 2018 accompanied by another beluga	^77^
5	Solitary Social Dolphin / Yera / Sally / Dolly / Beyoncé	New South Wales, Australia	Ta	F	Sept. 2012	No longer solitary	(9); ^40, 41^
0	?	Coffs Harbour, New South Wales, Australia	Ta	?	?	Present	G. Storrie 2018, pers. comm., 1 May
4 (5)	Stinky/Humpy/Randy	Gran Cayman	Tt	M	Approximately 2009	2012	^42, 43, 44^
4 (4)	Pechocho	Gulf of California, Mexico	Tt	M	Approximately 1992	Present	(10); ^1, 45^
4 (4)	Lucero	Veracruz, Mexico	Tt	F	Approximately 2003	Present	^46^
4 (4)^**^	Beggar / Mooch	Florida, USA	Tt	M	1990	Died 2012	(11); ^42^
4 (4)^**^	Dolphin 56	East Coast, USA	Tt	M	1979	Last seen Jul. 2011	^47, 48^
5	Kaimi	San Francisco, USA	Tt	?	Jul. 2016	No longer solitary	W. Keener 2018, pers. comm., 23 February; ^49^
4 (5)	Jojo	Turks & Caicos Islands, West Indies	Tt	M	1980	Present	(2, 5); ^50^
4 (?)	Moko	North Island, New Zealand	Tt	M	March 2007	Died Jun. 2010	I. Visser, 2018, pers. comm. 22 June; ^51, 52, 53^

* *Dl, Beluga (Delphinapterus leucas); Tt, Bottlenose dolphin (Tursiops truncatus); Ta, Indo-pacific bottlenose dolphin (Tursiops aduncus)*.

** *Interactions were limited to provisioning and touching*.

The corrected Table 2 and Table 3 appear below.

Additionally, there were also mistakes in the Footnotes. Incorrect links were published in Footnotes 12, 19, 56, and 67. The correct links for those footnotes appear below.

The authors state that this does not change the scientific conclusions of the article in any way. The original article has been updated.

